# Maternal vaccination with a type-III glycoconjugate protects mouse neonates against Group B Streptococcus intranasal infection

**DOI:** 10.1038/s41598-021-00941-z

**Published:** 2021-11-01

**Authors:** Emiliano Chiarot, Eleonora Naimo, Alessia Corrado, Patrizia Giannetti, Immaculada Margarit Y. Ros, Giuliano Bensi

**Affiliations:** 1 GSK Srl, Via Fiorentina 1, 53100 Siena, Italy; 2 Present Address: Hannover Medical School, Institute of Virology, Hannover, Germany; 3 Present Address: Bristol-Myers Squibb, 700 Bay Rd, Redwood City, CA 94063 USA

**Keywords:** Infectious diseases, Vaccines

## Abstract

Group B Streptococcus (GBS) is generally an asymptomatic colonizer of human mucosa but it occasionally infects pregnant women and neonates through vertical transmission, causing disease during the first weeks of life with frequent and severe complications. Preclinical studies have shown that maternal vaccination with polysaccharide-based vaccines protects mothers and offspring from GBS mucosal colonization and consecutive infection. In these models, bacteria were inoculated in mouse either intravaginally in the last trimester of pregnancy or systemically in pups. Here, we investigated whether maternal vaccination with glycoconjugate vaccines may also prevent GBS-mediated colonization and disease in neonates using an infection route that more closely mimics inhalation or ingestion of bacteria during human delivery. To address this point, mice aged less than two days were intranasally challenged with epidemiologically relevant GBS strains. Bacteria were found to colonize nose and intestine, reaching in some cases lungs and blood during the first days of life. Bacteria were also found in vagina of a fraction of colonized female mice within the first month of life. GBS-specific IgG induced by maternal vaccination with a glycoconjugate vaccine formulation were found in blood and mucosal tissues of newborns. Finally, when intranasally challenged with GBS serotype III strains, pups delivered by vaccinated mothers were partially protected against mucosal colonization and deeper infection.

## Introduction

Group B Streptococcus (GBS) is normally an asymptomatic member of the vaginal mucosa and lower gastrointestinal tract in up to 30% of pregnant women, but is also able to infect mothers, fetuses in utero and neonates, most likely by inhalation or/and ingestion of bacteria occurring during delivery^1–3^. Disease in the offspring may occur immediately after delivery or up to 6 months later, possibly leading to severe invasive manifestations including bacteremia, sepsis, pneumonia and meningitis^4,5^. When the symptoms occur within one week post-delivery it is defined as early onset disease (EOD) and as late onset disease (LOD) if later. Mucosal colonization, both in mothers and offspring, is considered one of the main risk factors for infection^6–11^. Six major GBS serotypes are responsible worldwide for up to 99% of EOD and LOD cases, but the most frequent one associated to LOD is serotype III and in particular ST-17 strains belonging to the clonal complex CC-17. These strains are often multi-drug resistant and hypervirulent and have been associated in some regions to almost 70% of LOD neonatal infections^12–15^.Intrapartum antibiotic prophylaxis (IAP) in women carrying GBS in the rectovaginal tract during the latest weeks of pregnancy resulted in a decrease of EOD incidence, but it did not affect colonization, maternal infection in previous stages of pregnancy or LOD^8,14,16^. Moreover, the emergence of antibiotic-resistant strains and possible adverse events for mothers and neonates underlined the need of different medical approaches, such as preventive vaccination^10,17–20^. Capsular polysaccharides conjugated to protein carriers have been recognized in the past decades as an effective vaccine against this pathogen, also because the combination of a few serotypes would guarantee the coverage of most GBS-dependent infections^19,21,22^. Preclinical models of GBS infection showed that these glycoconjugates could prevent maternal and offspring disease and reduce mucosal bacterial carriage^23,24^. However, in these models, bacteria were inoculated intravaginally in pregnant mice a few days before delivery or intraperitoneally in newborns, leaving open the question whether the vaccine had the capacity to confer protection to offspring against infection occurring during delivery when newborns can breathe or ingest bacteria. To address this question, we have developed a model of intranasal infection by bacterial inhalation in mouse neonates and we have followed bacterial spreading in several organs and tissues. GBS strains could colonize mucosal sites for at least one month post-infection and were transiently found also in lungs and blood. Remarkably, maternal vaccination with a polysaccharide type III glycoconjugate vaccine could protect mouse neonates against infection with a homologous GBS strain. A possible mechanism of action of the vaccine is proposed.

## Results

### Intranasal administration of GBS to mouse neonates results in long-lasting mucosal colonization

**Figure 1:**
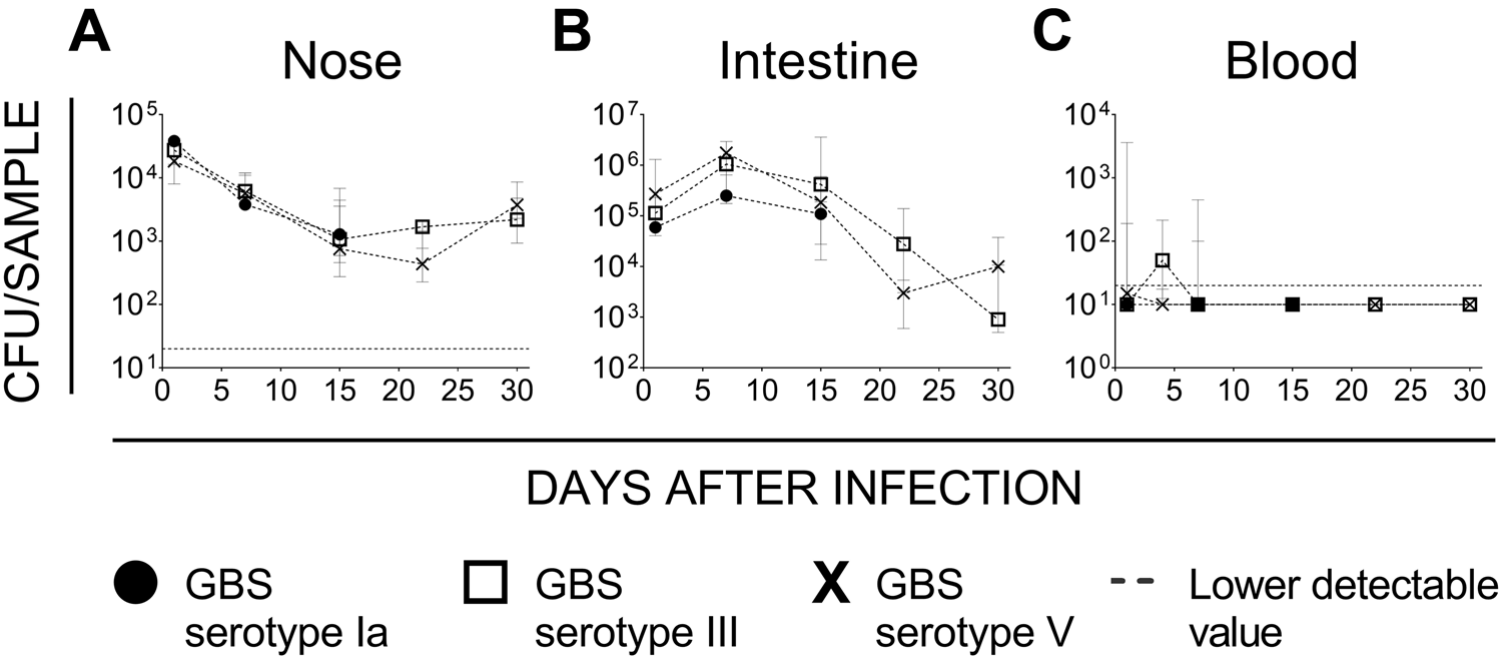
Intranasal infection with GBS resulted in long lasting colonization of nasal and intestinal mucosa and transient bacteremia in mouse neonates. Colony Forming Units (CFUs) enumerated during the time in nasal washes **(A)**, intestinal homogenates **(B)** and blood **(C)** of mouse pups intranasally infected with Group B Streptococcus (GBS) within the first two days of life. Three different GBS strains were tested belonging to serotype Ia (515), III (M781) and V (CJB111). Four to six samples were displayed at each time point from two/three independent experiments. Each dot represents the median value of the group and the bars depict 25 and 75 percentiles. In panels **(A,C)**, the dotted grey line is the lowest detectable value.

Inhalation of bacteria during delivery has been recognized as the preferred route of EOD infection in human neonates^25^. We wondered whether this condition could also be reproduced and used in preclinical murine models of neonatal disease. Independent groups of mouse pups aged less than two days were inoculated intranasally with 1–5 × 10^4^ colony-forming units (CFUs) of three epidemiologically relevant GBS strains (serotype Ia strain 515, serotype III strain M781 and serotype V strain CJB111) and then followed up to six weeks (15 days only for serotype Ia) to measure bacterial burden in nasal and intestinal mucosae as well as in lungs and blood. Between the 4th and the 6th week of life, vaginal swabs of GBS serotypes III and V infected females were also collected to check possible colonization. Bacteria were consistently found in nose and intestine during the first weeks following infection without significant differences among the three strains. While colonization of the nose decreased with time reaching a quantitative level of about 1 × 10^3^ CFU/wash in two weeks (Fig. 1A), colonization of the intestine peaked after seven days (1 × 10^6^ CFU/organ) and then slowly but progressively decreased with time (Fig. 1B). Importantly, spreading of bacteria to vaginal mucosa was also observed with type III and V strains (serotype Ia not tested, data reported in Table 1). GBS was also able to reach lungs, with bacteria being measured in 50–60% of pups during the first 15 days after infection, without evident peaks and major differences among the three serotypes (median values of the first two weeks of observation were 0.62 × 10^2^ CFU/organ for serotype Ia, 4.2 × 10^2^ CFU/organ for serotype III, 7.2 × 10^2^ CFU/organ for serotype V). Finally, only few animals experienced a transient bacteremia during the first days of life (Fig. 1C) and none of them died consecutively to the infection. In particular, the presence of bacteria in the blood of intranasally infected pups peaked within the first 7 days after infection for all the strains tested but with some differences. GBS serotype Ia bacteraemia peaked at day 7 (3 out of 8 pups), serotype III on day 4 (3 out of 4) but was still significantly present at day 7 (22 out of 47 pups), serotype V was found in blood of infected mice only at day 1 (3 out of 6 pups) and, partially, day 4 (1 out of 4).

**Table 1:** GBS vaginal colonization of intranasal infected animals occurred during the first weeks of life.

GBS serotype	Week after delivery	Tested animals—females (no.)	Positive samples (no.)
III	4th	8	1
V	4th	8	0
III	5th	11	0
V	5th	13	5
III	6th	10	2
V	6th	10	4

### Maternal immunization with CPSIII-CRM reduces GBS colonization and the risk of developing bacteremia in mouse pups

**Figure 2:**
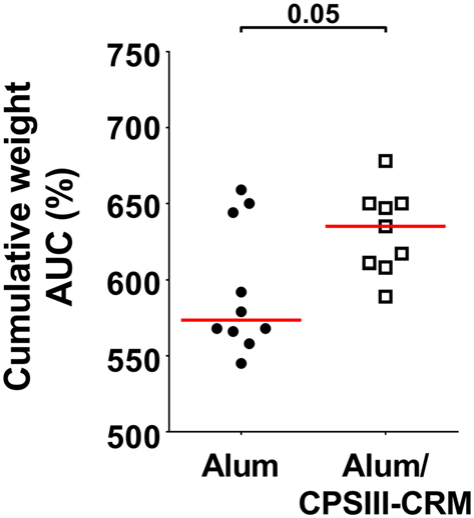
Maternal vaccination prevented weight loss in pups challenged with GBS M781 serotype III strain. Computed AUC (area under the curve) of normalized body weights of pups from alum/CPSIII-CRM- or alum-vaccinated mothers within the first 4 days following intranasal challenge with GBS M781 serotype III strain. Data were analyzed as increment of weight as compared with the initial weight of single animals (100%—normalized data) and finally AUC were calculated. Each dot represents the value of a single mouse. Data from 9 to 10 pups/group from one mother are reported. The Mann–Whitney U test was used to assess statistical significance.

**Figure 3:**
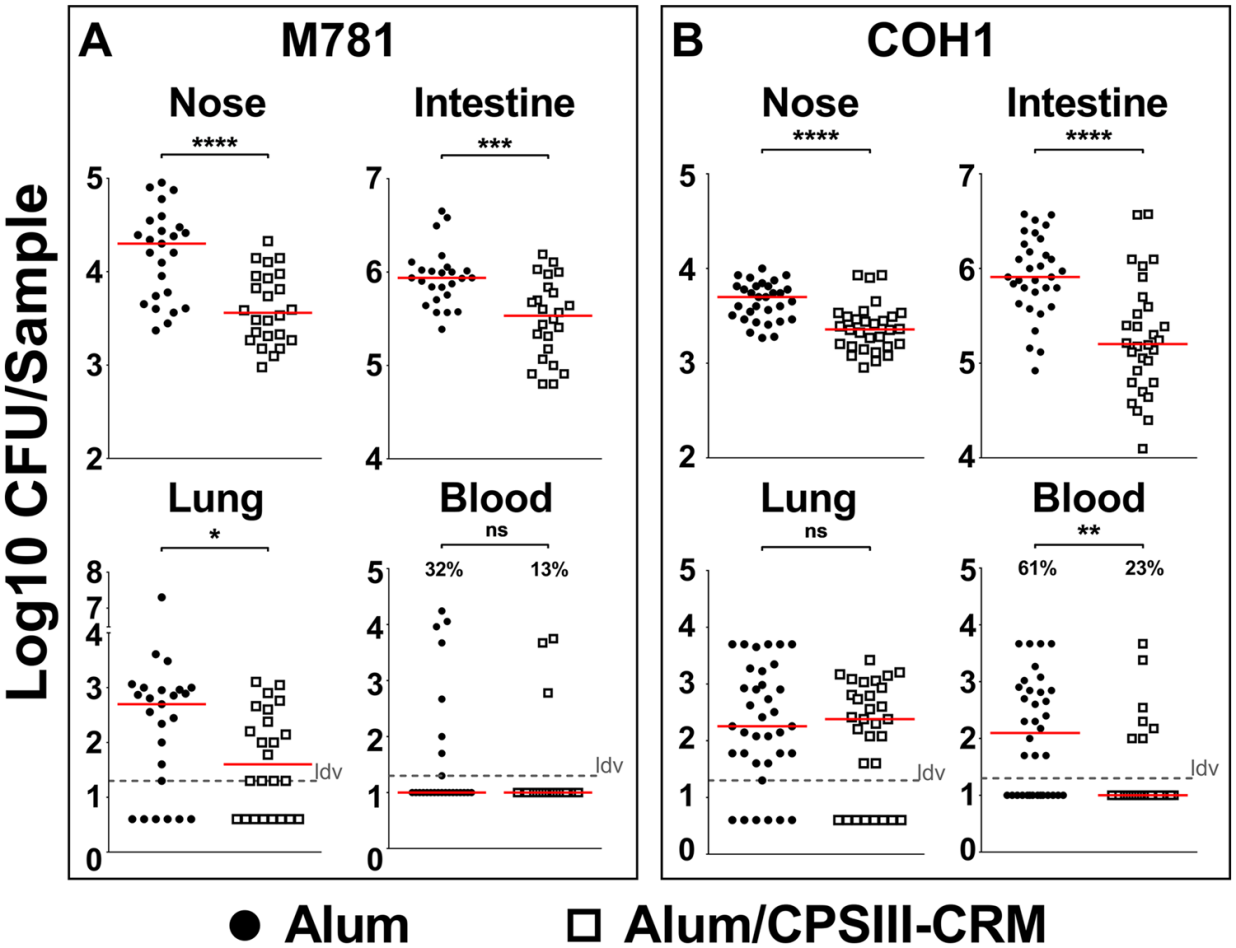
Pups from vaccinated mothers were protected against GBS colonization and deeper infection. CFU counts in nasal washes (Nose), intestinal homogenates (Intestine), lung and blood of mouse neonates from mothers immunized with alum/CPSIII-CRM or alum alone seven days after intranasal challenge with GBS serotype III strains M781 (**A**) or COH1 (**B**). Data from 24 to 30 mice/group and two–three independent experiments are reported for each strain tested. Each dot represents a single animal and the red horizontal line is the median value of the group. In the graphs reporting data from the blood, the percentage of pups experiencing bacteremia are also shown. When present, the grey dotted horizontal line depicts the lowest detectable value (ldv). *P < 0.05; **P < 0.01; ***P < 0.001; ****P < 0.0001 (Mann–Whitney U test or Fisher’s exact test for blood only).

**Figure 4:**
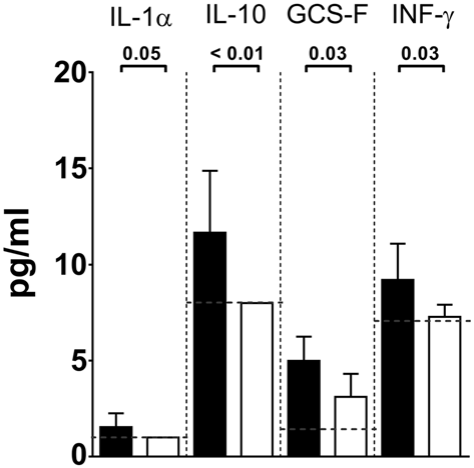
Maternal vaccination reduced systemic inflammation in pups after infection with serotype III GBS. Concentration of inflammatory cytokines (pg/ml) in the blood of infected pups seven days after challenge with GBS M781 serotype III strain. Data from 20–23 mice/group and three independent experiments are reported as geometric mean + 95% confidence intervals. Black bars report data from the negative control group (alum), white bars data from vaccinated group (alum/CPSIII-CRM). The lowest detectable value for each cytokine is represented by the grey dotted line. The Mann–Whitney U test was used to assess significance and the P value for each couple is reported above the graph.

After showing that mouse intranasal infection with GBS resulted in nasal and intestinal colonization and transient deeper infection, we aimed at using this model to assess protection induced by maternal vaccination. Female mice were intraperitoneally immunized three times with either GBS-type III capsular polysaccharide (CPS) conjugated with a detoxified form of diphtheria toxin (CRM_197_) and adjuvanted with aluminium hydroxide (alum/CPSIII-CRM) or with alum alone as negative control. After mating and delivery, pups were intranasally challenged with the GBS serotype III M781 (ST-17) strain within the following two days and their weight was measured daily for the following four days as a parameter reflecting the general health condition of the animals. When mice were born from vaccinated mothers, they grew significantly faster as compared with mice born from mothers vaccinated with adjuvant alone (Fig. 2). After seven days of observation, pups were euthanized to measure CFU counts and to perform serological analyses. Interestingly, as shown in Fig. 3 panel A, maternal vaccination with alum/CPSIII-CRM resulted in a lower colonization of nose and intestine and in a reduced bacterial burden in the lungs. Even if not statistically significant, as assessed by the Fisher’s exact test (P = 0.17), bacteria were found in the blood of only three out of 24 neonates from vaccinated mothers (13%) and in eight out of 25 pups from the control group (32%). To better elucidate whether vaccination could also be effective in preventing the likelihood to develop bacteremia and to confirm data obtained with the M781 strain, the hypervirulent type III COH1 strain (ST-17) was used to challenge pups and CFU in the blood as well as in mucosal sites and lungs were enumerated seven days after infection. As expected, a higher number of neonates developed bacteremia as compared to those infected with the M781 strain, but whereas bacteria were found in 7 out of 30 mice (23%) of the vaccinated group, 22 out of 36 (61%) neonates from non-vaccinated mothers were bacteremic (Fisher’s exact test; P = 0.0028, Fig. 3B). Moreover, a significant decrease of GBS colonization in the nose and intestine pups from vaccinated mothers was observed, while CPSIII-CRM/Alum vaccine did not reduce bacterial load in the lungs at the assessed time point (Fig. 3B). Additionally, when the pro-inflammatory status of mouse neonates was assessed by measuring the concentration of inflammatory cytokines in the blood of infected animals, a statistically significant lower concentration of inflammatory/anti-inflammatory mediators, such as interleukin (IL)-10, granulocyte colony-stimulating factor (G-CSF) and interferon (IFN)- was found in animals born from vaccinated mothers (Fig. 4).

### CPSIII-CRM maternal vaccination induces specific antibodies that are detected in sera and mucosa of newborns

**Figure 5:**
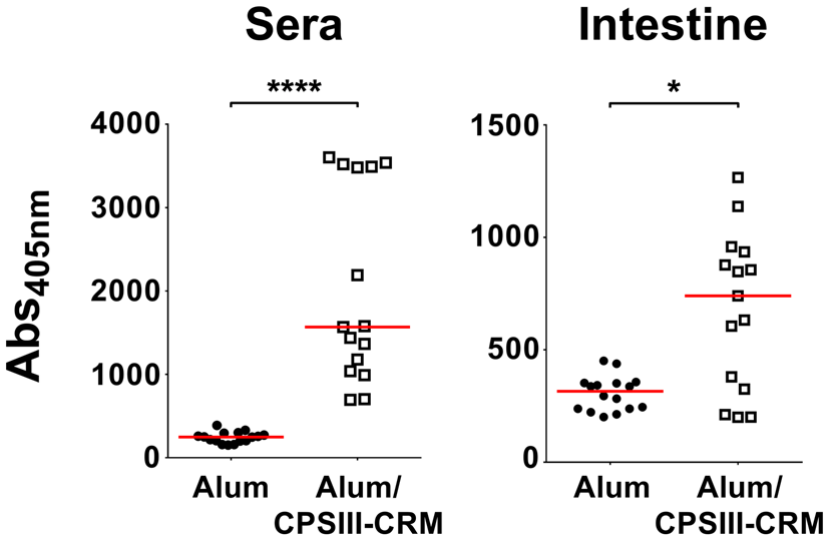
Antibodies against type III polysaccharide were found in blood and mucosal tissues of pups from vaccinated mothers. Anti-CPSIII IgG titres measured in the blood or intestinal homogenates (intestine) seven days after challenge with GBS are expressed as the intensity of absorbance at 405 nm. Each dot represents a single animal and the red horizontal line is the median value of the group. *P < 0.05; ****P < 0.0001 (Mann–Whitney U test).

The protection conferred to newborns could be explained by the induction in vaccinated mothers of type III polysaccharide-specific antibodies that are transferred to pups both through the placenta and with feeding^26^. To better support this hypothetical protection mechanism, an enzyme-linked immunosorbent assay (ELISA) was set up to measure type III polysaccharide-specific IgG in neonatal tissues and organs. Specific IgG against GBS polysaccharide III were found in both sera and homogenates of intestine collected from pups born from mothers vaccinated with alum/CPSIII-CRM (Fig. 5), but not in their nasal washes (data not shown). No CPSIII-specific antibodies were measured in pups born from mothers immunized with adjuvant only (Fig. 5).

## Discussion

Group B Streptococcus (GBS) is part of the normal commensal flora but is also recognized as a serious infectious threat especially during pregnancy and in the first weeks of life of neonates^27–29^. Chorioamnionitis, for example, is a common inflammatory disease during pregnancy, regardless of gestational week, and GBS carriage to the amniotic cavity has been recognized as one of the major risk factors^6,30–34^. Intrapartum antibiotic prophylaxis (IAP) has significantly decreased the number of maternal and neonatal infections that occur immediately before or after delivery but it has proven to be ineffective against mucosal colonization, late infections in neonates or maternal disease during the first trimesters of pregnancy^25,35^. Several evidences, both in humans and preclinical animal models, indicate that maternal vaccination may be an effective strategy to overcome these issues. For example, an inverse correlation was shown between anti-capsular antibody titres in mothers (and therefore in fetuses) and neonate infection, while vaccination with polysaccharide-based vaccines could significantly reduce the rate of non-protected mothers and offspring^36,37^. Moreover, when tested in humans, glycoconjugate vaccines showed an acceptable safety profile, were immunogenic and able to induce pro-opsonic antibodies^10,36,38–40^. Overall, these observations support their use as promising candidates and a 5/6-valent vaccine has been proposed to prevent around 99% of all perinatal infections, making its development extremely desirable^32,41^.

Preclinical rodent models of neonatal sepsis and perinatal diseases have also been used to show that maternal vaccination with glycoconjugate vaccines is able to protect both mothers and pups against GBS-mediated infection^23,42,43^. Interestingly, in a model of chorioamnionitis, mothers vaccinated with a GBS-type III polysaccharide conjugated to a protein carrier developed functional antibodies able to reach mucosal tissues and pregnant animals infected intravaginally a few days before delivery were protected against infection and mucosal carriage, as well as their fetuses and newborns^23^. What remained unclear was the effect of vaccination with glycoconjugate vaccines against neonatal infection that occurs during delivery.

The data presented here aimed at filling this gap. In the present manuscript, we report the use of a murine model in which pups were intranasally challenged early after delivery (most often within 24 h of life), better mimicking common conditions for infection which occur in humans^27,28^. For example, the delivery of bacteria was followed by a long-lasting carriage in the nose and in the intestine, which eventually evolved in vaginal colonization. Only half of the pups showed a deeper spread of bacteria (lungs and blood) and in all the cases this progression was transient and asymptomatic. The reason why the infection did not progress even if bacteria reached blood and lungs is still under investigation, but it may be the consequence of the very low bacterial load measured in these organs. Especially in the initial phases of colonization, we may envisage that bacterial spreading is actively counteracted by the reaction of the innate immune system, which could result in a positive outcome for the host if the bacterial load is initially too low to trigger the disease.

Pups from mothers vaccinated with the glycoconjugate vaccine were less susceptible to infection, showing an improved growth rate, a lower bacterial carriage at mucosal sites, in lungs and blood and a lower systemic inflammation. The latter observation should be pointed out since inflammatory conditions caused by GBS during pregnancy have been associated with neurological disorders of the offspring in preclinical models^44^. Another important result is the reduction of GBS colonization in neonates. Indeed, mucosal carriage has been reported to play a significant role in the development of LOD and therefore the reduction of colonization due to vaccination could also be an added value of this preventing approach as compared with IAP^11^. Specific IgG against GBS-polysaccharide have been found in sera and mucosae of protected pups, suggesting a possible functional role of these antibodies, as previously observed^23,45^. A possible effect against mucosal colonization of anti-capsule IgA induced by vaccination cannot be excluded. IgA could indeed be transferred from mothers to offspring as well as IgG^46^. Nevertheless, we do not expect that high levels of anti-capsule IgA were induced by intraperitoneal administration of an Alum-adjuvanted glycoconjugate vaccine and, therefore, if any, the possible role of specific IgA should not have played a pivotal role in mucosal protection of mouse pups^47–49^.

Taken together, the experimental data generated so far by using murine models support the hypothesis that use of glycoconjugate vaccines for maternal immunization at the beginning of pregnancy may significantly contribute to decrease the occurrence of maternal and neonatal GBS diseases.

## Methods

### Bacterial strains, growth conditions and preparation of CPSIII conjugates

GBS strains used for this work (type Ia, ST-23, strain 515; type III, ST-17, strain M781; type III, ST-17, strain COH1; type V, ST-1, strain CJB111^50^) were grown in liquid Todd-Hewitt broth (THB, BD) and plated onto Granada medium (BD). To start liquid cultures, frozen bacteria (15% glycerol in THB medium) were diluted in fresh THB at an initial absorbance of 600 nm (A_600_) = 0.05 in a standard 1-cm cuvette. They were then incubated statically at 37 °C without CO_2_ until reaching exponential phase (OD_600nm_ = 0.6), finally centrifuged (10 min at 3000 × *g* and 4 °C) and resuspended in fresh medium + 15% sterile glycerol to be stocked at − 80 °C (final concentration 3–4 × 10^8^ CFU/ml). The capsular polysaccharide III was extracted from GBS COH1, purified and randomly conjugated to CRM_197_ (CRM, detoxified diphtheria toxin) as previously described^51^.

### Ethical statements

Animal studies have been carried out following ARRIVE guidelines in an AAALAC accredited facility and in compliance with current Italian legislation on the care and use of animals in experimentation (Legislative Decree 26/2014) and with the GSK Vaccines Animal Welfare Policy and Standards. Protocols were approved by the Italian Ministry of Health (authorization DM292-2013B) and by the local GSK Vaccines Animal Welfare Body. Animals were caged in Individual Ventilated Cages (IVC) conditions with food and water ad libitum*.* Four-five mice were caged together until two days before delivery and then separated. Enrichment tools were used throughout all the experimental period. Sterile tap water was changed every seven days; cage and enrichment change was done every two weeks. In all the experiments, animals were monitored daily for the entire observation period and euthanized if they exhibited defined humane endpoints that had been pre-established for the study in agreement with GSK Vaccines Animal Welfare Policies.

### In vivo models of infection, immunization and protection

Glycerol stocks were diluted 1/10 in fresh medium and used to intranasally infect mouse neonates (< 2 day-old) CD1 mice (2 µl/nostril). Infective dose for each GBS strain was around 1.0–5.0 × 10^4^ CFU/mouse. To allow mice breathing the inoculum, they were slightly anesthetized for at least 10 min using isoflurane 1.5–2.0%. During this period, neonate body temperature was kept around 37° C . After infection, pups were housed again with the mothers and observed daily until they were euthanized. Males and females were separated 21 days after delivery and up to four (males) or five (females) animals were caged together. After euthanasia, nasal washes, intestine, lungs and blood were collected. All samples except blood and nasal washes were homogenized using gentleMACS Octo Dissociator-(Miltenyi Biotec) following supplier’s instructions. Blood was collected after beheading up to two weeks of age, then from the cheek. Nasal washes in pups were performed through the pharynx after removal of the lower jaw with 200 µl of PBS using a capillary inserted on a 200 µl tip. Vaginal swabs were performed in infected females 4–6 weeks after infection and diluted in 200 µl of PBS. For immunization experiments, five-week-old CD1 mice were injected three times intraperitoneally (200 µl) on days 0, 21 and 35. The vaccine was a glycoconjugate vaccine containing 1 µg of CPS-III conjugated with CRM197 and adjuvanted with 2 mg/ml aluminum hydroxide (alum). Females were then mated on day 38 after the first immunization and delivered pups were challenged within the first two days of life. Bleedings for collection of sera were performed, when necessary, two–three days before each immunization and serum was allowed to separate from the cellular part at room temperature for 4–6 h. Pups delivered from immunized mothers were weighted before infection and daily for four days after infection and weights were normalized based on the first measurement (100%) and plotted. Then the area under the curve (AUC) was computed based on the percentage of increase over time and data were reported as AUC for single animals. AUC from single animals was calculated using the following formula: ((normalized weight at time 0 + normalized weight at time 1) × (time 1 – time 0)/2) + ((normalized weight at time 1 + normalized weight at time 2) × (time 2 – time 1)/2) + ((normalized weight at time n + normalized weight at time n + 1) × (time n + 1 – time n)/2) etc.… Samples collected as described were plated on the selective Granada plates (BD) to count CFU. The remaining material was centrifuged at 21 000×*g* for 15 min to remove all the cellular debris and then filtered using a 0.22 µM filter. Sterilized samples were stored as previously described for ELISA and cytokine analysis^52^.

### ELISA for antigen-specific antibody

Microtiter 96-wells plate (NUNC, Maxisorp) were coated with 100 ng of GBS CPSIII conjugated to human serum albumin (CPS-III-HSA) via the spacer adipic acid dihydrazide in phosphate-buffered saline (PBS) pH 7.4^51^. Plates were incubated overnight at 2–8 °C, washed three times with PBST (0.05% Tween-20 in PBS pH 7.4) and saturated with 250 µl PBST-B (2% bovine serum albumin [BSA] in PBST) per well for 90 min at 37 °C. Sera from pups were tested at 1/1000 dilution, homogenates from intestine prepared as described above were tested undiluted. Plates were incubated at 37 °C for 1 h, washed with PBST, and then incubated for 90 min at 37 °C with anti-mouse IgG-alkaline phosphatase (Sigma, M8642) diluted 1:2000. After washing, the plates were developed with a 4 mg/ml solution of p-nitrophenyl phosphate (pNPP) in 1 M diethanolamine (DEA) pH 9.8, at room temperature for 30 min. After blocking with 7% w/v EDTA, the absorbance was measured using a SPECTRAmax plate reader with wavelength set at 405 nm. Absorbance measured in each single well was used as readout of the experiment.

### Cytokine analysis

Cytokine analysis was performed using the Bio-Plex Pro mouse cytokine standard 23-plex panel, group I (Bio-Rad) following manufacturer’s procedures. Sera samples from mice infected with M781 strain were prepared as described above. Reactions were read with the Luminex 200 system. Only cytokines that showed a statistically significant difference between glycoconjugate-vaccinated mice and control group were reported. List of cytokines analyzed: IL-1α, IL-1β, IL-2, IL-3, IL-4, IL-5, IL-6, IL-9, IL-10, IL-12p40, IL-12p70, IL-13, IL-17A, Eotaxin, G-CSF, granulocyte–macrophage colony-stimulating factor (GM-CSF), IFN-γ, cytokine‐induced neutrophil‐attracting chemokine (KC, equivalent to human IL-8), monocyte chemoattractant protein (MCP)-1, macrophage inflammatory protein (MIP)-1α, MIP-1β, Regulated upon Activation, Normal T Cell Expressed (RANTES), tumor necrosis factor (TNF)-α.

### Statistics

Statistical analyses were performed using the GraphPad Prism 7 software. The Mann–Whitney U-test (two tailed) or the Fisher’s exact test (two tailed) were used as reported in figure legends to calculate statistical significance. P values < 0.05 were considered statistically significant. Legend: *P < 0.05; **P < 0.01; ***P < 0.001; ****P < 0.0001.

## Data Availability

The datasets generated and/or analyzed during the current study are available from the corresponding author on reasonable request.
